# Central Serous Chorioretinopathy Deteriorated With Everolimus Administration in Advanced Renal Cell Carcinoma: A Case Report

**DOI:** 10.1155/crop/1200325

**Published:** 2026-04-17

**Authors:** Mahdi Nemati, Mohammadreza Niyousha, Banafshe Kharazi, Narges Hassanpoor

**Affiliations:** ^1^ Retina & Vitreous Service, Nikookari Eye Hospital, Tabriz University of Medical Sciences, Tabriz, Iran, tbzmed.ac.ir; ^2^ Student Research Committee, Tabriz University of Medical Sciences, Tabriz, Iran, tbzmed.ac.ir

**Keywords:** central serous chorioretinopathy, CSCR, everolimus, pachychoroid, renal cell carcinoma

## Abstract

**Background:**

Everolimus, an antineoplastic drug, is associated with various systemic adverse effects. This case report is aimed at presenting a novel ophthalmological complication, drug‐associated central serous chorioretinopathy (CSCR), following everolimus administration.

**Methods:**

A 54‐year‐old male with metastatic renal cell carcinoma presented with blurred vision and metamorphopsia. Comprehensive ophthalmologic examination, including multimodal imaging with optical coherence tomography (OCT), fluorescein angiography (FA), and fundus autofluorescence (FAF), was performed. A thorough review of his medical history and recent medication changes was conducted to identify potential associating factors.

**Results:**

Multimodal imaging confirmed bilateral CSCR with subretinal fluid and pachychoroid features. The only recent change in his medication regimen was the initiation of everolimus (10 mg daily) 1 month prior to symptom onset. All other known risk factors for CSCR, such as corticosteroid use, were absent. Upon discontinuation of everolimus, the patient′s symptoms and visual acuity improved. Follow‐up OCT imaging demonstrated complete resolution of the subretinal fluid.

**Conclusion:**

Everolimus can potentially be a risk factor for subclinical CSCR deterioration in prone population. Ophthalmologists and oncologists should be aware of this rare but significant adverse effect.

## 1. Introduction

Everolimus is an oral mammalian target of rapamycin (mTOR) inhibitor that affects cell proliferation, survival, and angiogenesis. It is primarily used in cancer treatment and other conditions where the mTOR pathway plays a crucial role. Everolimus has several therapeutic uses across various medical conditions, including advanced renal cell carcinoma (RCC), neuroendocrine tumors, and breast cancer. It also acts as an immunosuppressive therapy to prevent organ rejection in kidney and liver transplant, rare genetic disorders like Tuberous Sclerosis Complex (TSC), and other rare conditions like cardiac sarcoidosis. [1–4] Everolimus is an important treatment option for metastatic RCC, particularly in patients who have failed first‐line targeted therapies. A Phase 3 trial demonstrated its superiority over placebo, highlighting its effectiveness in this context. [5]

Despite its high efficacy in advanced RCC, everolimus is known for significant adverse effects namely fatigue, rash, stomatitis, anemia, leukopenia, bleeding diathesis, and fetal adverse events, most of which thoroughly observed and reported in multiple meta‐analysis studies. [6–10] One of the noteworthy rare reported side effects of everolimus is generalized edema, which was explained in kidney transplant patients receiving the drug. Cessation of everolimus is advised to be considered if there is progressively increasing proteinuria, especially if > 3 g/day, or if associated with peripheral edema. [11–13]

Reported ophthalmologic adverse effects, however, are significantly less common and mostly include eyelid edema and conjunctivitis, with their mechanism of action yet to be explained. [12, 13] In the current study, we report the first documented case, to the best of our knowledge, of everolimus‐associated central serous chorioretinopathy (CSCR) deterioration in a patient with advanced RCC.

## 2. Case Presentation

A 54‐year‐old male known case of advanced RCC was referred to our center for recent central vision loss, micropsia, and metamorphopsia of 3 weeks′ duration. Ophthalmologic examination revealed 20/30 best corrected visual acuity (BCVA) in right and 20/40 in left eye. Relative afferent pupillary defect was negative in both eyes. Intraocular pressure values were 15 and 18 mmHg. Anterior segment examination revealed no significant abnormality with cornea and anterior chamber being free of any possible inflammatory response. During posterior segment examination, absence of normal tessellation that indicates an underlying pachychoroid and decreased foveal reflex were noticed in both eyes (Figure 1).

FIGURE 1Fundus photos of the (a) right and (b) left eye showing an orange‐reddish appearance and decreased foveal reflex. Fundus autofluorescence (FAF) of the (c) right and (d) left eye showing areas of hypo and hyperautofluorescence possibly revealing an asymptomatic stage of CSCR being clinically evident following everolimus consumption. Fluorescein angiogram (FA) of the (e–g) right eye shows window defect (red arrow) and expansile dot leakage (blue arrow). FA of the (h–j) left eye shows two expansile dot leakages (blue arrow).

**Figure:**
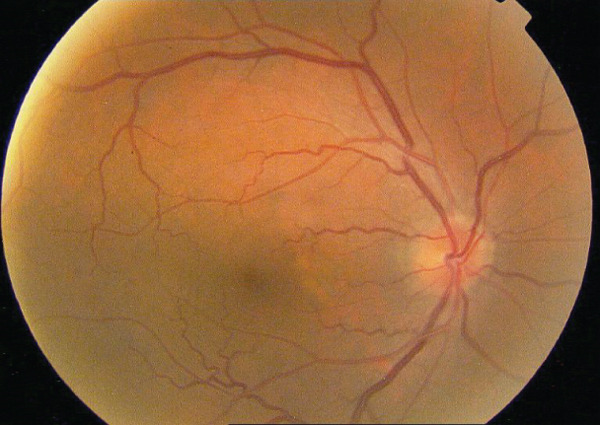
(a)

**Figure:**
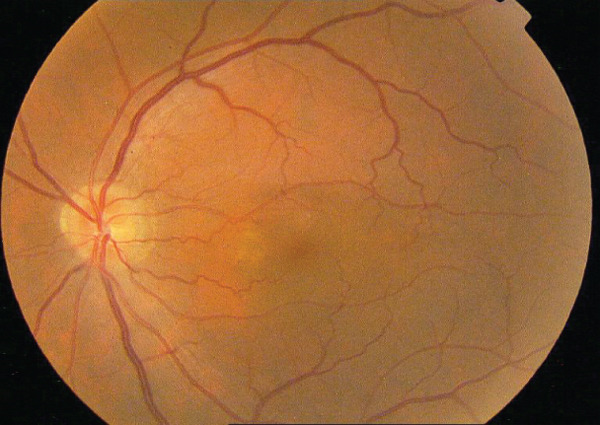
(b)

**Figure:**
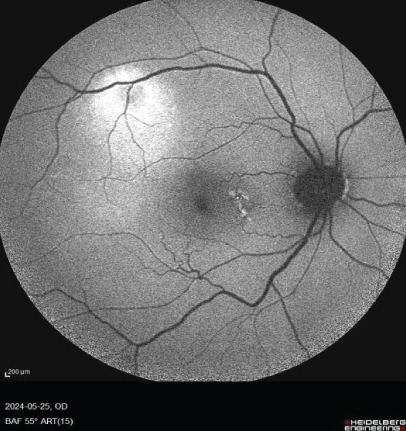
(c)

**Figure:**
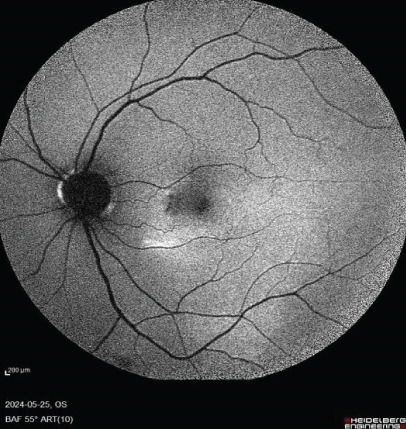
(d)

**Figure:**
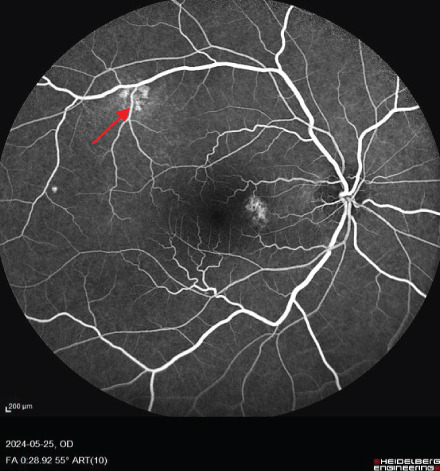
(e)

**Figure:**
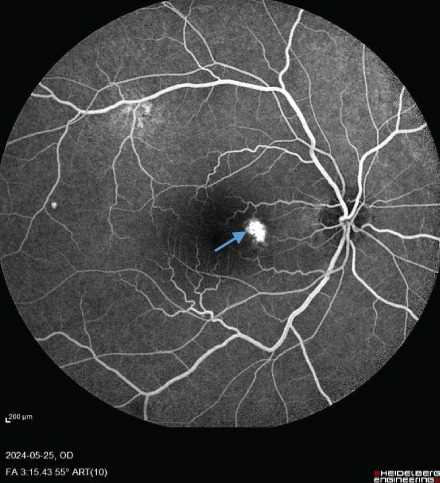
(f)

**Figure:**
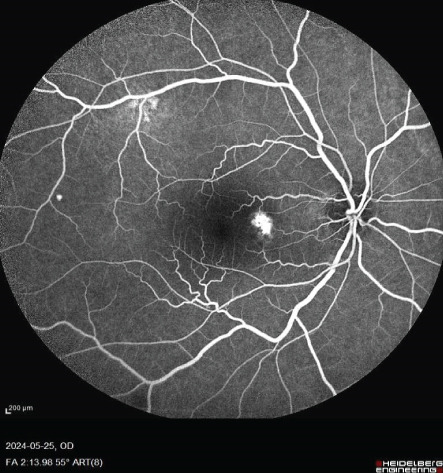
(g)

**Figure:**
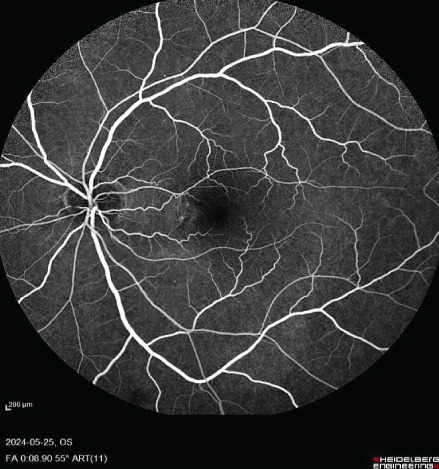
(h)

**Figure:**
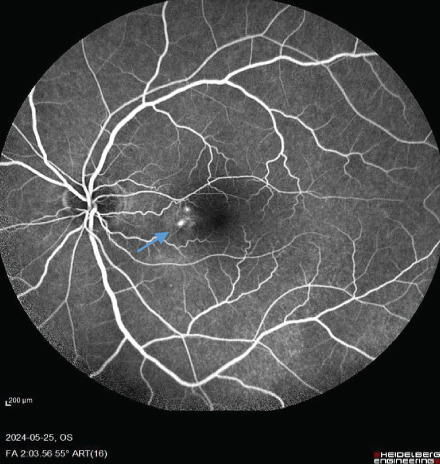
(i)

**Figure:**
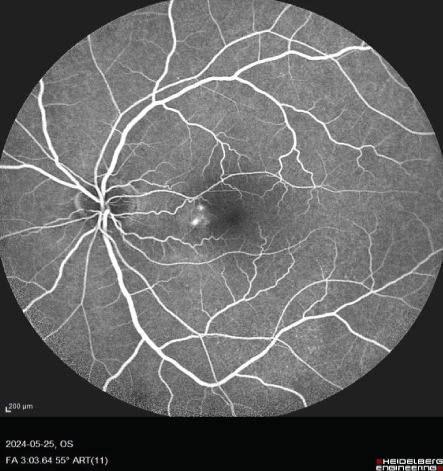
(j)

Multimodal imaging consisting of optical coherence tomography (OCT), OCT angiography (OCTA), fundus autofluorescence (FAF), near infrared (NIR), and fluorescein angiography (FA) images was obtained and analyzed. In his right eye OCT, thick choroid with pachyvessels, subretinal fluid, and a serous pigment epithelial detachment (PED) are obvious. Left eye OCT shows foveal subretinal fluid overlying a thick choroid with pachyvessels (Figure 2).

FIGURE 2Optical coherence tomography (OCT) of the right and left eye (a and b) at presentation, (c and d) 1 month, and (e and f) 2.5 months after everolimus discontinuation. In his (a) right eye OCT, thick choroid with pachyvessels, subretinal fluid, and a serous pigment epithelial detachment (PED) are obvious. (b) Left eye OCT shows foveal subretinal fluid overlying a thick choroid with “pachyvessels.” (e and f) OCT changes resolved with mild outer retinal atrophy after 2.5 months of everolimus discontinuation.

**Figure:**
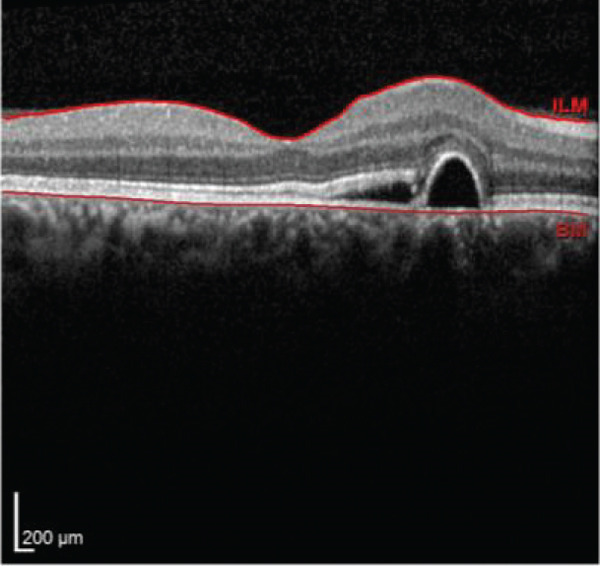
(a)

**Figure:**
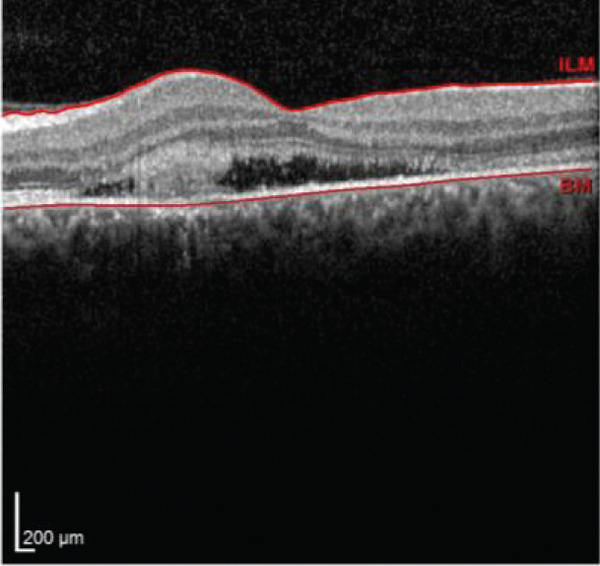
(b)

**Figure:**
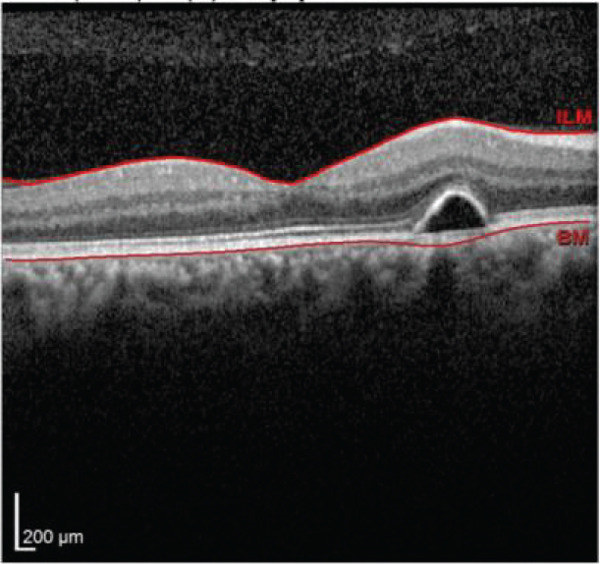
(c)

**Figure:**
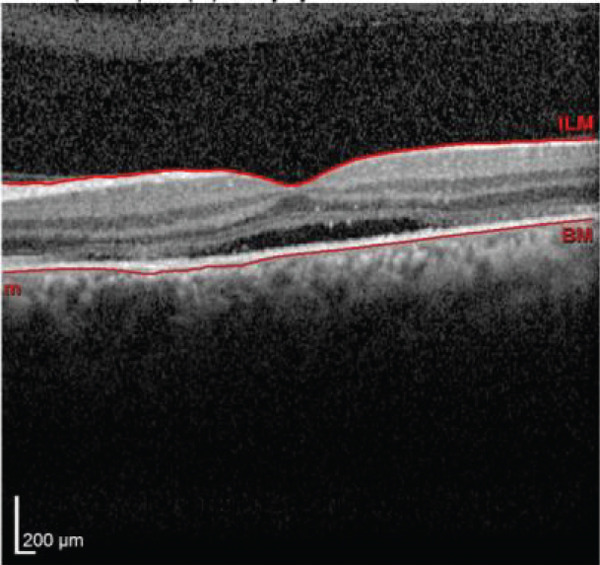
(d)

**Figure:**
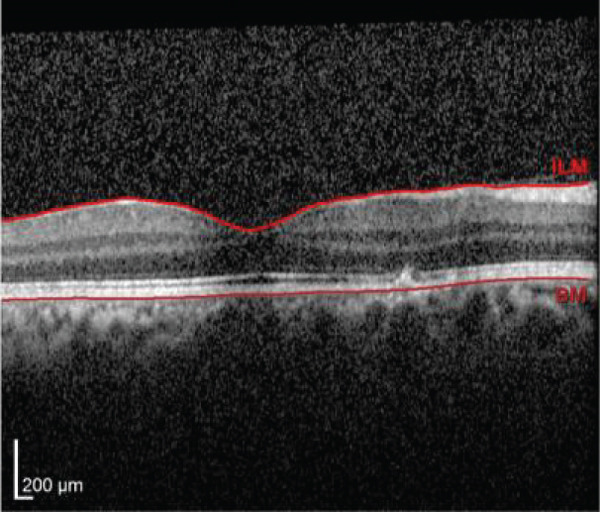
(e)

**Figure:**
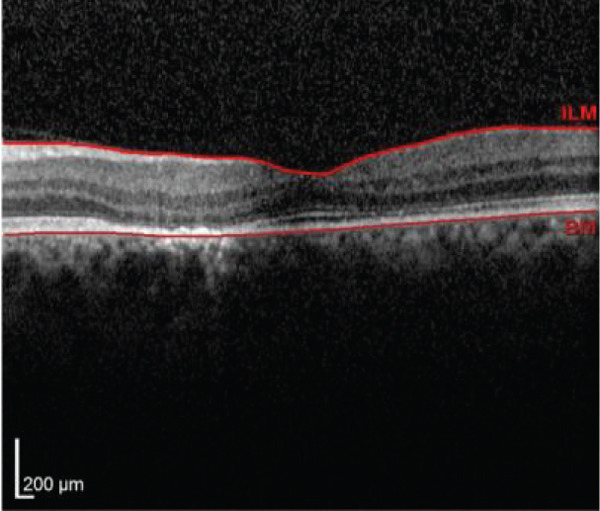
(f)

He was diagnosed with CSCR. In comprehensive risk factor evaluation and history taking, there were neither previous history of similar visual symptoms nor recent systemic or topical steroid prescription. Medical history also affirmed no recent change in other medical treatment regimen or drugs newly prescribed in the past 4 months other than everolimus with the dosage of 10 mg daily orally from 1 month ago. Patient stated that symptoms have gradually been worsened during a course of 3 weeks previous to the ophthalmology visit. After counseling with his uro‐oncology specialist, administration of everolimus was discontinued. Patient signs and symptoms were being monitored for around 3 months. He demonstrated a good response to everolimus cessation, with BCVA improving to 20/25 in both eyes during this follow‐up period. He also mentioned significant improvement of his symptoms of metamorphopsia and micropsia even in the first follow‐up 2 weeks after drug cessation. Ophthalmologic examination and multimodal imaging also revealed resolution of serous PED and subretinal fluid with mild outer retinal atrophy in his left eye (Figure 2). Further follow‐up conducted 5 months later revealed clinically stable findings and no decrease in BCVA values.

### 2.1. Methods and Statistical Analysis

This is a single case report. Descriptive statistics are used to present the clinical data. The primary analyses were the qualitative assessment of multimodal imaging findings and the clinical course following drug cessation. All analyses were prespecified as observational assessments of the patient′s clinical progress. No formal hypothesis testing was performed, and thus no a priori significance level was set for statistical tests. All imaging analyses were performed using the built‐in software of the Spectralis HRA + OCT system (Heidelberg Engineering, Germany).

### 2.2. Ethical Statement

Written informed consent was obtained from the patient for both the procedure and the publication of his case details and images. The study adhered to the tenets of the Declaration of Helsinki.

## 3. Discussion

CSCR, presenting as the fourth common reason for retinopathy, is capable of causing significant central vision distortion. [14] Although first noticed in 1866 by Albrecht von Graefe, its pathogenesis is yet to be fully understood with most hypothesis currently supporting choroidal circulatory autoregulation dysfunction causing hyperpermeability of tissue and thus subretinal fluid accumulation. [15, 16] Koizumi et al. recently found that thicker sclera in patients with CSCR may contribute to choroidal circulatory disturbances. The increased scleral thickness could lead to higher resistance in vortex veins and reduced choroidal venous outflow, which may negatively impact choroidal circulation. They also suggested the term “pachysclera” instead of pachychoroid. [17] The two‐hit theory in CSCR, as discussed by Koizumi et al., posits that anatomical predispositions such as vortex vein asymmetry, scleral thickening, and short axial length interact with environmental triggers including stress, steroids, or other medications that influence the mineralocorticosteroid pathway. These interactions may lead to increased vortex vein congestion, which can exacerbate extravascular leakage and fluid accumulation in subretinal space. [17] This theory highlights the importance of both predisposing and precipitating factors leading to the disease.

Drug‐induced CSCR is a known complication of mitogen‐activated protein kinase (MEK) and fibroblast growth factor receptor (FGFR) inhibitors currently being consumed in cases of advance staged cancers. [18] Methylenedioxymethamphetamine also known as ecstasy and sildenafil have also been reported to cause subretinal fluid accumulation with unknown mechanisms yet to be detected. [19, 20]

Everolimus is an oral derivative of sirolimus, a selective inhibitor of the mTOR pathway. It has shown promising antineoplastic activity and is used in various cancer treatments. [1–3] Although everolimus has many adverse effects on numerous organs of human body, its known ophthalmological side effects are rather less common, as periorbital edema and conjunctivitis being the top two mostly encountered side effects. Despite reports of these adverse effects in literature, the exact mechanism responsible for fluid accumulation has not been discovered. [21, 22] This edema of periorbital tissue may have possible shared mechanism as one occurring in our CSCR suffering patient. One possible mechanism could be everolimus induced proteinuria and subsequent fluid accumulation in various tissues. [11–13] The other possible mechanism can be related to everolimus effects on mineralocorticosteroid and androgen pathways, which has been proved to play part in CSCR pathophysiology as increased level of serum aldosterone and androgen hormones may mediate fluid flow in choroidal vessels.

It has been shown that unlike other mTOR inhibitors, everolimus does not have antimineralocorticosteroid effect seen in other mTOR inhibitors like rapamycin, with no decrease in serum aldosterone levels indicating its possible role in mediating fluid accumulating hormone effects on various tissues such as ocular choroidal vessels. Furthermore, it has been speculated that everolimus may play a role as an androgen receptor upregulating agent in cancerous cells showing its possible contribution in androgen fluid retention effects in ocular vascular tissues as other tissues of body. This androgen receptor elevation effect has not been detected by other mTOR inhibitors like sirolimus. [23, 24]

In this case, history taking, examination, reviewing current medications, and any recent alteration on drug regimen combined with multimodal imaging plus resolution of subretinal fluid following drug cessation further supported this medication deteriorated CSCR. Everolimus may act as a risk factor for CSCR in individuals with pre‐existing conditions, such as a pachychoroid phenotype or subclinical CSCR, rather than directly causing it. However, as CSCR can be a self‐limiting disorder, the possibility of self‐resolution cannot be ruled out. While the application of mTOR inhibitors is becoming more widespread, acknowledging potential side effects, particularly ophthalmological complications, is crucial for managing patient care. Future reports and studies may provide additional insights into these effects and help optimize the use of mTOR inhibitors in clinical practice.

**Nomenclature**
RCC: renal cell carcinoma
CSCR: central serous chorioretinopathy
BCVA: best corrected visual acuity
OD: oculus dexter
OS: oculus sinister
PED: pigment epithelial detachment
mTOR: mammalian target of rapamycin inhibitor
OCT: optical coherence tomography
OCTA: optical coherence tomography angiography

## Author Contributions

Narges Hassanpoor had full access to all of the data in this study and takes complete responsibility for the integrity of the data and the accuracy of the data analysis.

## Funding

No funding was received for this manuscript.

## Disclosure

All authors have read and approved the final version of the manuscript. Narges Hassanpoor affirms that this manuscript is an honest, accurate, and transparent account of the study being reported; that no important aspects of the study have been omitted; and that any discrepancies from the study as planned have been explained.

## Consent

Written informed consent for publication of clinical details and/or images has been obtained from the patient.

## Conflicts of Interest

The authors declare no conflicts of interests.

## Data Availability

The data that support the findings of this study are available on request from the corresponding author. The data are not publicly available due to privacy or ethical restrictions.
